# Enhancement of Inhibitory Activity by Combining Allosteric Inhibitors Putatively Binding to Different Allosteric Sites on Cathepsin K

**DOI:** 10.3390/molecules28104197

**Published:** 2023-05-19

**Authors:** Shun Sato, Kana Yamamoto, Moeno Ito, Katsutoshi Nishino, Takanao Otsuka, Kazuhiro Irie, Masaya Nagao

**Affiliations:** 1 Graduate School of Biostudies, Kyoto University, Kyoto 606-8502, Japan; 2 Department of Applied Chemistry and Biotechnology, Okayama University of Science, Okayama 700-0005, Japan; 3 Graduate School of Agriculture, Kyoto University, Kyoto 606-8502, Japan

**Keywords:** cathepsin K, allosteric, inhibitor, pheophytin *a*, pheophorbide *b*, *Chamaecrista nomame*

## Abstract

Background: Cathepsin K, which is involved in bone resorption, is a good target for treating osteoporosis, but no clinically approved medicine has been developed. Recently, allosteric inhibitors with high specificity and few side effects have been attracting attention for use in new medicines. Methods: Cathepsin K inhibitors were isolated from the methanol extract of *Chamaecrista nomame* (Leguminosae) using cathepsin K inhibition activity-assisted multi-step chromatography. Standard kinetic analysis was employed to examine the mechanism of cathepsin K inhibition when an isolated inhibitor and its derivative were used. The allosteric binding of these cathepsin K inhibitors was supported by a docking study using AutoDock vina. Combinations of allosteric cathepsin K inhibitors expected to bind to different allosteric sites were examined by means of cathepsin K inhibition assay. Results: Two types of cathepsin K inhibitors were identified in the methanol extract of *Chamaecrista nomame*. One type consisted of cassiaoccidentalin B and torachrysone 8-β-gentiobioside, and inhibited both cathepsin K and B with similar inhibitory potential, while the other type of inhibitor consisted of pheophytin *a*, and inhibited cathepsin K but not cathepsin B, suggesting that pheophytin *a* binds to an allosteric site of cathepsin K. Kinetic analysis of inhibitory activity suggested that pheophytin *a* and its derivative, pheophorbide *b*, bind allosterically to cathepsin K. This possibility was supported by a docking study on cathepsin K. The cathepsin K inhibitory activity of pheophytin *a* and pheophorbide *b* was enhanced by combining them with the allosteric inhibitors NSC 13345 and NSC94914, which bind to other allosteric sites on cathepsin K. Conclusions: Different allosteric inhibitors that bind to different sites in combination, as shown in this study, may be useful for designing new allosteric inhibitory drugs with high specificity and few side effects.

## 1. Introduction

Cathepsins are proteases found in lysosomes; cathepsin K (Cat K), a lysosomal cysteine protease that degrades type I collagen, is involved in bone resorption [1], while cathepsin B (Cat B) is also a cysteine protease in a cathepsin family that plays a role in inflammation, for example, by promoting the processing and secretion of interleukin-1β (IL-1β) [2]. Osteoporosis is a chronic bone disease cause by an imbalance between bone formation and bone resorption. Alendronate, a bisphosphonate, and Denosumab, an anti-receptor activator of nuclear factor κB ligand (RANKL) antibody, have been approved by the Food and Drug Administration (FDA) as a clinical medicine for osteoporosis [3]. Although Cat K inhibitors could be effective as anti-osteoporosis drugs, none have yet been approved by the FDA. Odanacatib (ODN), a Cat K-selective oral inhibitor, has entered into clinical trials, but the development of ODN as an anti-osteoporosis drug was stopped because of its adverse cardio-cerebrovascular effects [4].

We screened various plant extracts for inhibitory activity against Cat K. An extract of the aerial part of *Chamaecrista nomame* (Leguminosae), which is commonly used as a herbal tea, and which is grown in China and Japan—especially Kochi Prefecture—showed inhibitory activity against Cat K. Here, we report the two types of Cat K inhibitors we identified: the first type inhibited both Cat K and Cat B, and the other type inhibited Cat K but not Cat B, and is expected to function as an allosteric inhibitor of Cat K. We examined whether a combination of allosteric inhibitors binding to different allosteric sites had a higher inhibitory effect.

Since the FDA approved the use of two allosteric inhibitors, asciminib, which inhibits Breakpoint cluster region–Abelson1 (BCR-ABL1) tyrosine kinase [5], and deucravacitinib, which inhibits tyrosine kinase 2 (TYK2) [6], the clinical application of allosteric inhibitors has recently started attracting attention. Allosteric inhibitors have advantages over orthosteric inhibitors that bind to active sites, such as greater specificity and reduced side effects [7].

Combining allosteric inhibitors that bind to different allosteric sites on Cat K is suggested to be a useful tactic for the development of new allosteric inhibitors in clinical medicine.

## 2. Results

### 2.1. Identification of Cathepsin K Inhibitors in the Extract of Chamaecrista Nomame and Analysis of Specificity of Cathepsin K Inhibitors, including Pheophytin a Derivatives

#### 2.1.1. Purification of Cat K Inhibitors from the Methanol Extract of *Chamaecrista nomame*

**Figure 1:**
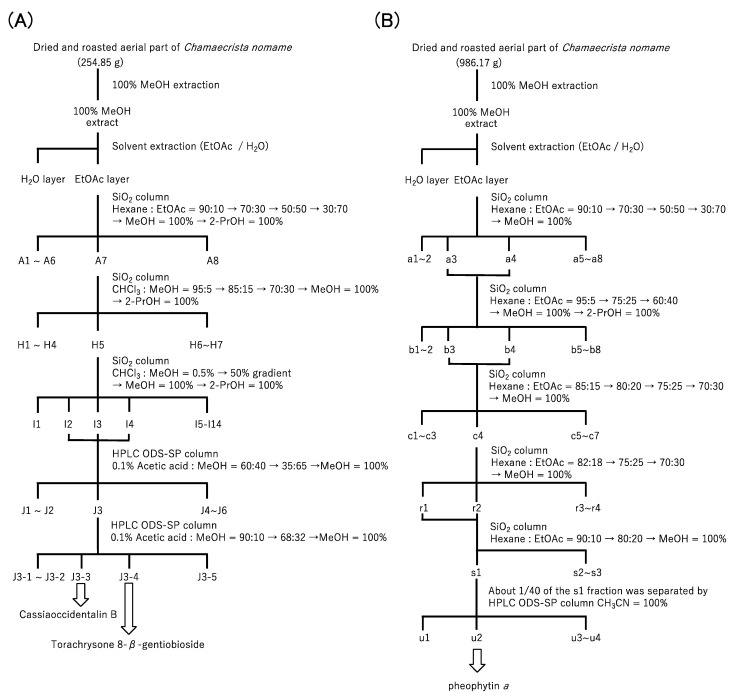
Schematic diagrams of the purification of Cat K inhibitors from the roasted aerial part of *Chamaecrista nomame* on a small scale (**A**) and on a large scale (**B**).

The procedure for the purification of Cat K inhibitors from the roasted aerial part of *Chamaecrista nomame* is shown in Figure 1.

#### 2.1.2. Identification of Two Types of Cat K Inhibitor in the Methanol Extract of *Chamaecrista nomame* and Their Inhibitory Activity towards Cat K and B

**Figure 2:**
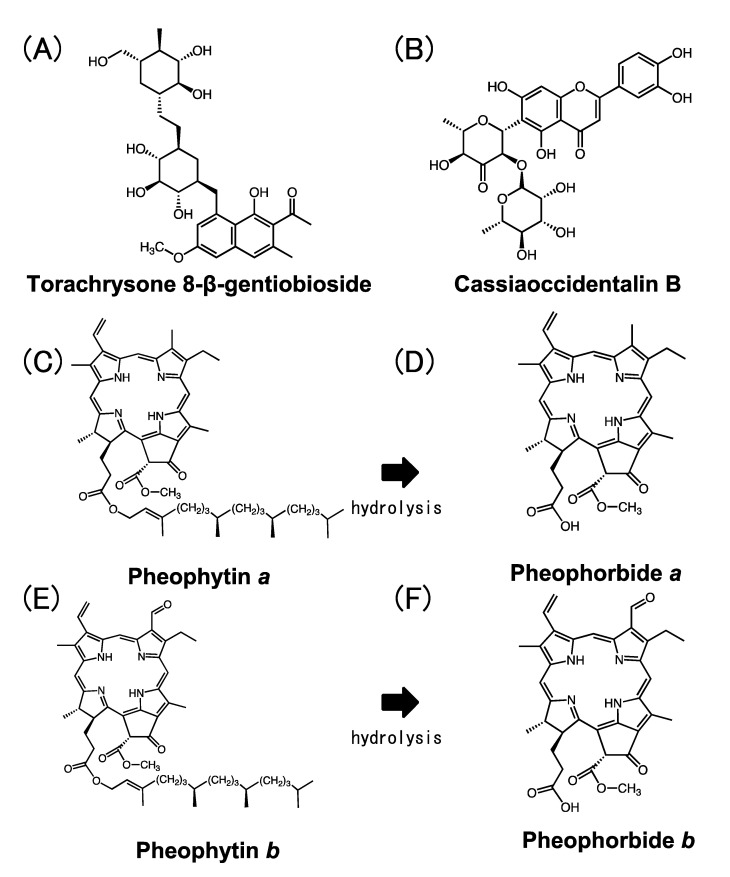
Chemical structure of inhibitors of Cat K isolated from the extract of *Chamaecrista nomame* (**A**–**C**) and pheophytin *a* derivatives (**D**–**F**): (**A**) torachrysone 8-β-gentiobioside; (**B**) cassiaoccidentalin B; (**C**) pheophytin *a*; (**D**) pheophorbide *a*; (**E**) pheophytin *b*; (**F**) pheophorbide *b*.

**Figure 3:**
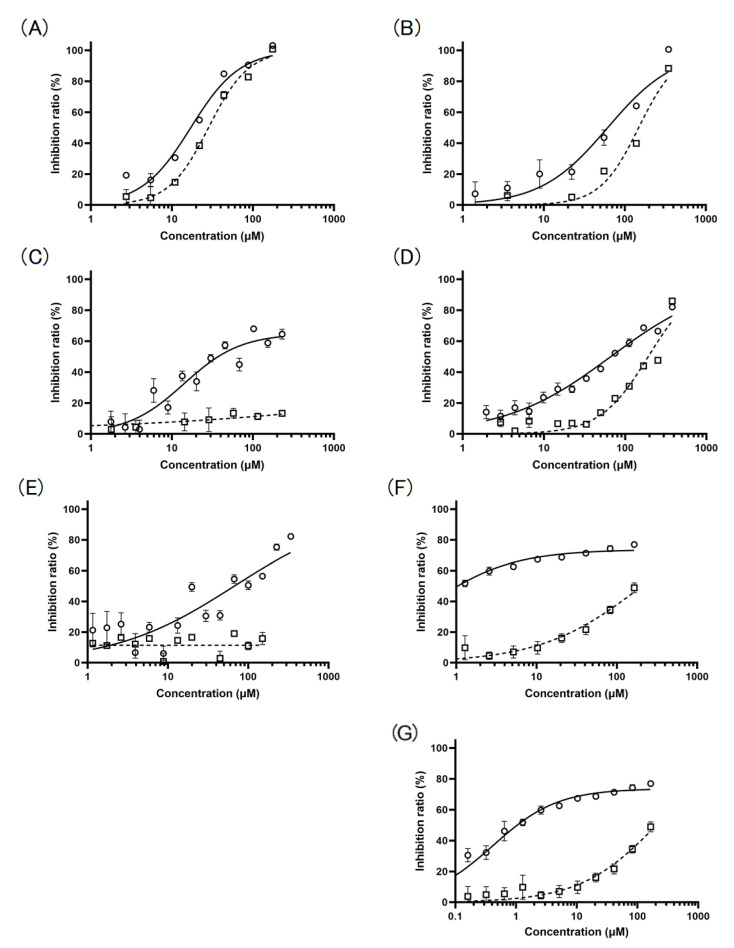
Dose-dependent inhibition of Cat K (open circle, solid line) and Cat B (open square, dotted line) by the inhibitors described in Figure 1: (**A**) torachrysone 8-β-gentiobioside; (**B**) cassiaoccidentalin B; (**C**) pheophytin *a*; (**D**) pheophorbide *a*; (**E**) pheophytin *b*; (**F**) pheophorbide *b*; (**G**) pheophorbide *b* (wider range in concentration). Results are presented as means ± SE (*n* = 3). GraphPad Prism 9.5.1 was used for curve fitting. In the calculation of curve fitting, maximal inhibition was set at 100%.

Three Cat K inhibitors, whose NMR spectra were identical to those reported for torachrysone 8-β-gentiobioside [8], cassiaoccidentalin B [9] and pheophytin *a* [10], were identified as Cat K inhibitors in the methanol extract of *Chamaecrista nomame* (Leguminosae) (Figure 2A–C). LC-MS analyses of these compounds confirmed their chemical structures. Torachrysone 8-β-gentiobioside and cassiaoccidentalin B inhibited both Cat K (IC_50_ = 17. 1 ± 1.07 μM and 61.6 ± 1.17 μM, respectively) and B (IC_50_ = 28.4 ± 1.06 μM and 148.5 ± 1.12 μM, respectively), with similar inhibitory potential (Figure 3A,B), while pheophytin *a* inhibited Cat K (IC_50_ = 14.1 ± 1.23 μM) but not Cat B (Figure 3C).

Pheophorbide *a* and pheophorbide *b* were prepared by hydrolysis of the phytyl residue of pheophytin *a* and pheophytin *b*, respectively, isolated from the extract of spinach.

#### 2.1.3. Preparation of Pheophytin *a* Derivatives and Their Inhibitory Activity towards Cat K and Cat B

Pheophytin *a* and pheophytin *b* were isolated from the methanol extract of the aerial part of spinach, as reported previously [10], and pheophorbide *a* and pheophorbide *b* were prepared by acid hydrolysis of pheophytin *a* and pheophytin *b* (Figure 2C,E), respectively, using trifluoroacetic acid (Figure 2D,F). The chemical structures of these derivatives of pheophytin *a* were confirmed by NMR analyses (see NMR data in the Supplementary Materials). Pheophorbide *a*, a breakdown compound of pheophytin *a*, in which the phytol (phytyl group) was removed by hydrolysis at the acetyl group of C17^3^ position (Figure 2D), inhibited Cat K more weakly (IC_50_ = 66.6 ± 1.07 μM) than pheophytin *a*, and weakly inhibited Cat B (IC_50_ = 192.8 ± 1.06 μM) (Figure 3D), while pheophytin *a* did not inhibit Cat B (Figure 3C). Pheophytin *b*, a compound in which the methyl group at the C7^1^ position of pheophytin *a* was converted to an aldehyde group (Figure 3E), inhibited Cat K (IC_50_ = 67.3 ± 1.20 μM) slightly more weakly than pheophytin *a*, but had no inhibitory activity towards Cat B (Figure 3E). Pheophorbide *b*, in which the phytyl group at the acetyl group of the C17^3^ position of pheophytin *b* was removed (Figure 2F), inhibited Cat K (IC_50_ = 0.42 ± 1.16 μM) more strongly than pheophytin *a*, but only weakly inhibited Cat B (IC_50_ = 206.6 ± 1.24 μM) (Figure 3F,G).

### 2.2. Mode of Cathepsin K Inhibition by Pheophytin a and Pheophorbide b and Determination of Kinetic Parameters

#### 2.2.1. Allosteric Inhibition of Cat K by Pheophytin *a* and Pheophorbide *b*

**Figure 4:**
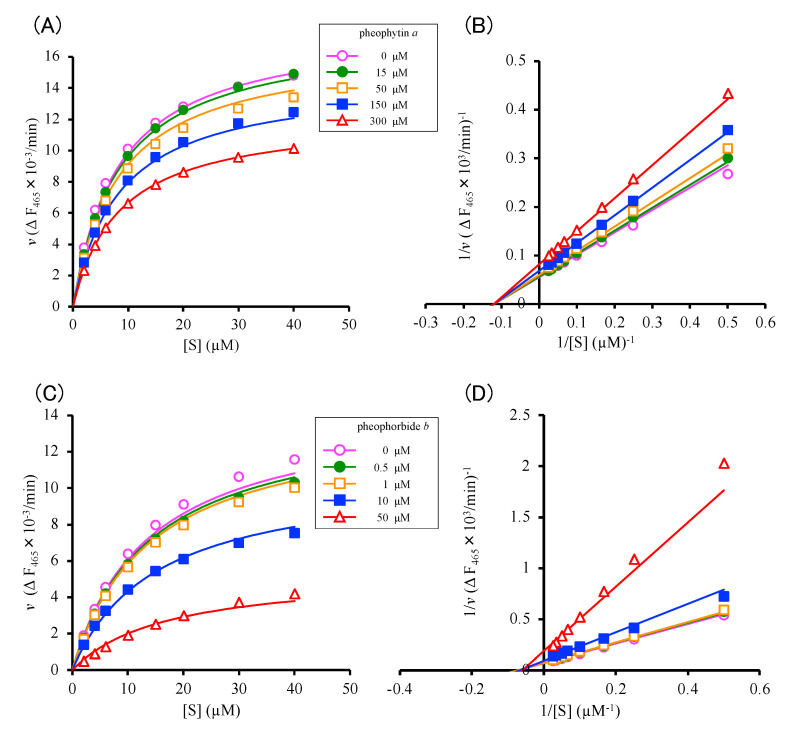
Inhibition of Cat K activity by pheophytin *a* and pheophorbide *b*. Eight concentrations of substrate ((**A**,**B**); 2, 4, 6, 10,15, 20, 30, and 40 μM) and five concentrations of inhibitors ((**A**) 0 (open circle in pink), 15 (closed circle in green), 50 (open square in orange), 150 (closed square in blue), 300 (open triangle in red) μM of pheophytin *a* and (**B**) 0 (open circle in pink), 0.5 (closed circle in green), 1 (open square in orange), 10 (closed square in blue), 50 (open triangle in red) μM of pheophorbide *b*). The kinetic parameters were calculated by curve fitting the experimental data to the non-competitive inhibition equation for pheophytin *a* and mixed type inhibition equation for pheophorbide *b* [11]. Lineweaver–Burk plots for the inhibition of Cat K by (**C**) pheophytin *a* and (**D**) pheophorbide *b* were used to determine the inhibition mode.

Pheophytin *a* specifically inhibited Cat K, but not Cat B (Figure 3C), and pheophorbide *b* inhibited Cat K, but only very weakly inhibited Cat B (Figure 3F,G). The inhibition modes and the inhibition constants of the two compounds for Cat K were determined in the presence of various concentrations of pheophytin *a* and pheophorbide *b* (Figure 4A,C). According to the Lineweaver–Burk plots, the mode of inhibition of pheophytin *a* was non-competitive, and that of pheophorbide *b* was of a mixed type (Figure 4B,D). The inhibition constant calculated for pheophytin *a* was 630 ± 21.2 μM (K_i_). The inhibition constant calculated for pheophorbide *b*, which was expressed as the inhibitory parameters between the enzyme and the inhibitor, K_ic_, and that between the enzyme–substrate complex and the inhibitor, K_iu_, and were 21.6 ± 4.4 μM (K_ic_), and 29.3 ± 5.2 μM (K_iu_), respectively [11]. Similar K_m_ values were obtained in the two assays (8.32 ± 0.20 μM for pheophytin *a* and 13.9 ± 0.8 μM for pheophorbide *b*). Thus, both pheophytin *a* and pheophorbide *b* were considered to act as allosteric inhibitors against Cat K.

### 2.3. Analysis of Allosteric Binding of Pheophytin a and Pheophorbide b Using AutoDock Vina

#### 2.3.1. Docking Simulation of Pheophytin *a* and Pheophorbide *b* to Cat K

**Figure 5:**
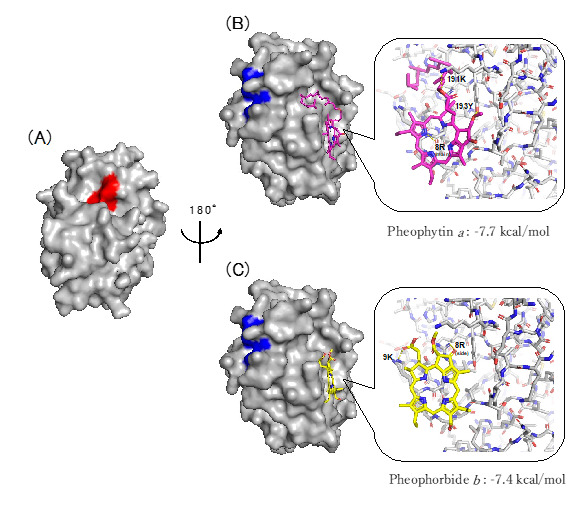
Speculated binding sites of pheophytin *a* and pheophorbide *b* to Cat K (PBD code 6QL8). (**A**) The amino acids composing the active center of cathepsin K are shown in red (S24, C25, W26, N161, and H162). (**B**,**C**) The common amino acids involved in the binding of NSC13345 and NSC94914 to Cat K reported previously (K122, R198, and N199) [14,15,16] are shown in blue on the opposite side of Cat K. The active site of cathepsin K (red) is on the front side of Cat K (**A**). The binding sites of pheophytin *a* (magenta) (**B**) and pheophorbide *b* (yellow), as well as (**C**) the common binding sites of NSC13345 [14] and NSC94914 [15,16] (blue) (**B**,**C**), are located on the back side of Cat K. The grid sizes were set to 68 × 54 × 50 points and 60 × 50 × 54 points for pheophytin *a* (**B**) and pheophorbide *b* (**C**), respectively, with a spacing of 1.0 Å. The docking scores −7.7 kcal/mol and −7.4 kcal/mol against Cat K for pheophytin *a* (**B**) and pheophorbide *b* (**C**), respectively, were calculated using AutoDock vina. Amino acids 8R, 191K and 193Y in Cat K are speculated to be involved in the binding to pheophytin *a*, and amino acids 8R and 9K in that to pheophorbide *b*.

The docking simulation of the binding of pheophytin *a* and pheophorbide *b* to Cat K using AutoDock vina [12,13] suggested that these compounds bind not to the active center of Cat K, colored in red, but to the allosteric sites, as shown in Figure 5. The kinetic analysis of the inhibition of Cat K by pheophytin *a* and pheophorbide *b*, described in Section 2.2.1, suggested that pheophytin *a* and pheophorbide *b* inhibited Cat K in a non-competitive and mixed inhibitory fashion, binding, respectively, not to the active center, but to allosteric sites on Cat K (Figure 5), which supports these results.

#### 2.3.2. Pheophytin *a* and Pheophorbide *b* Bind to Each Allosteric Site That Is Different from the Allosteric Sites on Cat K, to Which the Known Allosteric Inhibitors NSC13345 and NSC94914 Bind 

The known allosteric inhibitors of Cat K, NSC13345 [14,17] and NSC94914 [15,16], were found to bind to the sites shown in blue in Figure 5, and these sites were different from the allosteric sites to which pheophytin a and pheophorbide b were supposed to bind, according to the analysis performed using AutoDock vina (Figure 5).

### 2.4. Additional Inhibition of Cat K by a Combination of Allosteric Inhibitors That Bind to Different Allosteric Sites

**Figure 6:**
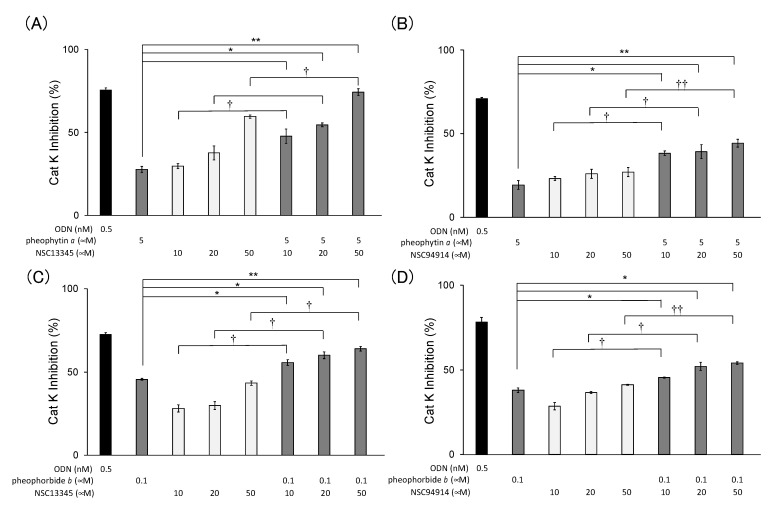
Additive inhibition of Cat K by pheophytin *a* and pheophorbide *b* with NSC13345 and NSC94914. Inhibition of Cat K activity by pheophytin *a* and pheophorbide *b* with and without NSC13345 and NSC94914 was examined. Odanacatib (ODN), a known Cat K inhibitor, was used as a positive control. Results are presented as mean ± SD (*n* = 3). * *p* < 0.05, ** *p* < 0.01 vs. pheophytin *a* only (**A**,**B**) and pheophorbide *b* only (**C**,**D**). † *p* < 0.05, †† *p* < 0.01 vs. without pheophytin *a* (**A**,**B**) and pheophorbide *b* (**C**,**D**) by Student’s *t*-test.

Since NSC13345 and NSC94914 bind to each closely related allosteric site to which pheophytin *a* and pheophorbide *b* do not bind (Figure 5A–C), we attempted to obtain an additional inhibitory effect by combining allosteric inhibitors between NSC13345 and NSC94914 with pheophytin *a* and pheophorbide *b*. On the basis of preliminary experiments, the doses used in combination with NSC13345 or NSC94914 were 5 μM for pheophytin *a* and 0.1 μM for pheophorbide *b*. The inhibitory activity of pheophytin *a* at 5 μM against Cat K was enhanced by the addition of 10 to 50 μM of either NSC13345 or NSC94914 (Figure 6A,B). The inhibitory activity of pheophorbide *b* at 0.1 μM was similarly enhanced by addition of 10 to 50 μM of NSC13345 or NSC94914 (Figure 6C,D). On the other hand, the dose-dependent inhibition of cathepsin K by NSC13345 and NSC94914 was enhanced by the addition of pheophytin *a* at 5 μM and pheophorbide *b* at 0.1 μM (Figure 6A–D).

## 3. Discussion

Osteoporosis is a bone erosive disease caused by imbalance of bone resorption and bone formation mediated by osteoclasts and osteoblasts, respectively. Natural plant-derived products have been reported to be effective against bone erosive diseases [18]. Cat K, a lysosomal cysteine proteinase highly expressed in the osteoclasts and involved in bone resorption by degrading type I collagen, the major components of the bone matrix [1], is a good target to consider for treatment of osteoporosis. Among several Cat K inhibitors examined for activity against osteoporosis, odanacatib is a prospective candidate, proposed for clinical use, but it has been found to have adverse cardio-cerebrovascular effects [4].

We found inhibitory activity against Cat K in several plant extracts, including the extract of *Salvia officinalis*, from which we isolated aryl hydrocarbon receptor ligands [19], and that of *Perilla frutescens* var. *crispa*, a culinary herb also used in Chinese medicine. In this study, two types of Cat K inhibitor were identified from the extract of *Chamaecrista nomame*. The first type consisted of cassiaoccidentalin B and torachrysone 8-β-gentiobioside, and it inhibited both Cat K and Cat B (Figure 3A,B), while the second type consisted of pheophytin *a*, which bound allosterically to Cat K (Figure 4B and Figure 5A,B) and inhibited Cat K only (Figure 3C). Cassinodentalin B isolated from *Cassia glauca* has been reported to inhibit α-amylase and α-glucosidase [20], suggesting that cassinodentalin B is an inhibitor of multiple enzymes, including Cat K. Cassinodentalin B isolated from *Cymbopogon citratus* has been reported to have potential anti-inflammatory activity [21]. Torachrysone 8-β-gentiobioside isolated from *Cassia tora* (Leguminosae) has been reported to show anti-bacterial activity against methicillin-resistant *Staphylococcus aureus* [8], but this is the first report that cassinodentalin B and torachrysone 8-β-gentiobioside inhibit Cat K. Pheophytin *a*, which only inhibited Cat K, is a chlorophyll *a* lacking a central Mg^2+^ ion. The expected allosteric binding of pheophytin *a* to Cat K deduced by the docking study supported a rationale for the specificity of pheophytin *a* to Cat K, but not to Cat B (Figure 5). Pheophytins and pheophorbides derived from chlorophyll perform photosynthesis, and can be utilized for photodynamic therapy (PDT) [22,23]. The photoactive properties of chlorophyll derivatives, such as pheophytin and pheophorbide, which generate singlet oxygen in the presence of light, have potential in PDT, such as for use in cancer therapy [22]. Four chlorophyll derivatives, pheophytin *a*, pheophytin *b*, pheophorbide *a* and pheophorbide *b*, have shown similar anti-cancer activities, but pheophytin *a* has the weakest activity and pheophorbide *a* and *b* have the strongest activity against hepatoma Huh7 cells in PDT [22].

Allosteric inhibitors are drawing attention as safer alternatives to active site-directed inhibitors such as the allosteric inhibitors of Cat K, NSC13345 [14,17] and NSC94914 [15,16]. In this study, pheophytin *a* was identified as a possible allosteric inhibitor of Cat K without inhibiting Cat B. Pheophorbide *b*, a derivative of pheophytin *a* that was expected to bind allosterically to Cat K, exhibited stronger inhibitory activity against Cat K than pheophytin *a*, but exhibited weak inhibitory activity against Cat B. Pheophytin *a* and pheophytin *b* exhibited inhibitory activity against Cat K, but not against Cat B (Figure 3C,E), while pheophorbide *a* and pheophorbide *b* exhibited inhibitory activity against both Cat K and Cat B (Figure 3D,F), suggesting that the phytyl group bound at the C17^3^ position of pheophytin *a* and pheophytin *b* preserves the inhibitory activity against Cat B. Why pheophorbide *b*, whose phytyl group at the C17^3^ position of pheophytin *a* is removed and whose methyl group at the C7^1^ position of pheophytin *a* is converted to an aldehyde group, has higher inhibitory activity than pheophytin *a* (Figure 3C,F,G) remains to be examined.

Pheophytin is a derivative of chlorophyll whose Mg^2+^ ion is dechelated from its porphyrin ring (Figure 2C,E). Porphyrin is utilized as a surface module that attaches to the protein–protein interaction (PPI) interface to design a PPI modulator that has an anchor portion that grips a protein, for example, by some ionic interaction, attached to a porphyrin scaffold [24,25]. The aldehyde group at the C7^1^ position of pheophorbide *b*, but not of pheophorbide *a*, may function as such an anchor attached to the porphyrin ring, enhancing the inhibitory activity against Cat K more than pheophytin *a*.

The mode of the inhibitory activity exerted by pheophytin *a* and pheophorbide *b* was evaluated using the Lineweaver–Burk linear transformation technique, and it was suggested that pheophytin *a* inhibited Cat K in a non-competitive manner, while pheophorbide *b* exhibited a mixed manner of inhibition (Figure 3C,D). Both compounds were supposed to bind to Cat K in an allosteric manner, and the binding sites of pheophytin *a* and pheophorbide *b* were different from those of the known allosteric Cat K inhibitors NSC13345 and NSC94914 bind [14,15,16,17] (Figure 5). We used AutoDock vina for the docking study, although “DIFFDOCK” (https://openreview.net/pdf?id=kKF8_K-mBbS accessed on 12 April 2023) may be useful for increasing the accuracy of the docking study. In the present case, we examined the additive effects of combining these allosteric inhibitors (Figure 6). The combination of the allosteric inhibitors pheophytin *a* and pheophorbide *b* with NSC13345 or NSC94914 enhanced their inhibitory activity against Cat K. If a covalent inhibitor that binds to the substrate pocket of Cat K that blocks the binding of ODN, but not pheophytin *a* or pheophorbide *b*, is found, the allosteric binding of pheophytin *a* and pheophorbide *b* will be confirmed. Recently, the combination of osimertinib, an ATP-binding competitive inhibitor of epidermal growth factor receptor (EGFR), with EGFR allosteric inhibitors was reported as a next-generation lung cancer therapeutic strategy [26]. Allosteric inhibitors are receiving increased attention, since allosteric inhibitors are expected to offer high specificity with only minor side-effects [27]. Very recently, the allosteric inhibitors asciminib, which inhibits BCR-ABL1 tyrosine kinase [5], and deucravacitinib, which inhibits tyrosine kinase 2 (TYK2) [6], were approved by the FDA for use in clinical medicine. Incidentally, there have been several reports concerning the synergistic effect of allosteric ligands that bind to the allosteric sites of same protein target, such as peroxisome proliferated receptor ligands [28]. The combination of non-competitive allosteric inhibitors binding to different allosteric sites on Cat K may be a useful tactic for designing more effective enzyme inhibitory drugs in clinical medicine.

## 4. Materials and Methods

### 4.1. Chemical Reagents and Instruments

Organic solvents for fractionation and DTT, EDTA and DMSO were purchased from Nacalai Tesque, Kyoto, Japan. Triton-X100 was purchased from Fujifilm Wako Pure Chemical, Osaka, Japan. NSC13345 (2-[(2-carbamoylsulfanylacetyl)-amino]benzoic acid) and NSC94914 ((2-biphenylylmethyl)malonic acid)) were obtained from the US NCI/DTP Open Chemical Repository (Washington, DC, USA). ^1^H and ^13^C NMR spectra were measured and recorded on Avance III 400 and 500 (reference TMS, Burker, Bremen, Germany). High-resolution ESI-MS data were obtained on a Waters ACQUITY^TM^ UPLC with Xevo G2-S QTof mass spectrometer (Nihon Waters, Shinagawa, Japan).

### 4.2. Preparation of Extract of Chamaecrista nomame and Purification of Cat K Inhibitors

The dried and roasted aerial parts of *Chamaecrista nomame* (254.85 g and 986.17 g), obtained from a local farmer in Kochi prefecture in Japan, were extracted with methanol (3.4 L and 7.9 L) for one week at room temperature. After filtration, filtrates were evaporated until dry in vacuo at 40 °C to afford methanol extracts (46.89 g and 130.05 g). The purification steps of the extracts are described in Figure 1A,B. The methanol extracts were partitioned between ethyl acetate and H_2_O. Silica gel column chromatography (Wako gel C-200, Fujifilm Wako Pure Chemical Corporation, Osaka, Japan; YFLC AI-580, Yamazen Corporation, Osaka, Japan) and HPLC (Chromaster, Hitachi High-Tech Science Corporation, Tokyo, Japan) using ODS-SP (GL Sciences, Osaka, Japan) were employed for further purification, as shown in Figure 1A,B.

### 4.3. Preparation of Pheophytin a Derivatives from Extract of Spinach

The aerial part of spinach was purchased from a local market in Kyoto. Pheophytin *a* and pheophytin *b* were prepared according to the method described in a previous report [10]. Pheophorbide *a* and Pheophorbide *b* were prepared by hydrolysis of pheophytin *a* and pheophytin *b*, respectively, in the presence of trifluoroacetic acid.

### 4.4. Measurement of Inhibitory Activity of Inhibitors against Cat K and Cat B

Recombinant human procathepsin K was purchased from ENZO Life Science, Farmingdale, NY, USA and activated according to the manual. Activity of Cat K was measured in 50 mM acetate buffer, pH 5.5, 1 mM DTT, 2.5 mM EDTA, 0.1% Triton-X100, 1% DMSO, and 30 µM z-Phe-Arg-AMC (Peptide Institute, Osaka, Japan) as a substrate. Prior to addition of the substrate, an inhibitor was preincubated for 10 min at room temperature with enzyme to allow the establishment of the enzyme–inhibitor complex. A substrate was then added, and after incubation for 20 min at 37 °C, the enzyme activity was measured from the increase in fluorescence at 465 nm (λex = 360 nm). Assays were performed using a 384-well microplate (Corning^®^ 384 well Low Volume Black Round Bottom Polystyrene NBS Microplate, Product Number 4514), and fluorescence was measured using a Power Scan 4 plate reader (DS Pharma Biomedical, Suita, Japan). Inhibition ratio was calculated using the following equation:

(1) $$\mathrm{Inhibition}\;\mathrm{ratio}\;\left(\%\right)=\left(1-\frac{\left\{F\left(\mathrm{sample}+E+S\right)-F\left(\mathrm{sample}\right)\right\}-F\left(\mathrm{blank}\right)\}}{F\left(E+S\right)-F\left(\mathrm{blank}\right)}\right)\times 100$$

F(x): Fluorescent intensity (excitation at 360 nm, emission at 465 nm); E: enzyme; S: substrate; sample: inhibitor sample; blank: buffer.

Human liver Cat B was purchased from Sigma Aldrich (St. Louis, MO, USA). The activity of Cat B was measured in 50 mM acetate buffer, pH 5.5, 1 mM DTT, 2.5 mM EDTA, 0.1% Triton-X100, 1% DMSO, and 30 µM z-Arg-Arg-AMC (Peptide Institute, Osaka, Japan) as a substrate. Prior to the addition of substrate, an inhibitor was preincubated for 10 min at room temperature with enzyme to allow the establishment of the enzyme–inhibitor complex. The substrate was then added, and after incubation for 20 min at 37 °C, the enzyme activity was measured on the basis of the increase in fluorescence at 465 nm (λex = 360 nm). Assays were performed using a 384-well microplate.

Drawing curves of inhibition and calculation of IC_50_ were executed by GraphPad Prism 9.5.1 (GraphPad, San Diego, CA, USA).

### 4.5. Kinetic Analysis of Cat K Inhibition

For kinetic studies of Cat K, the inhibition assay was carried out as described in Section 4.4. The substrate concentrations were fixed at 2, 4, 6, 10,15, 20, 30, and 40 μM, while the inhibitor concentrations were 0, 15, 50, 150 and 300 µM for pheophytin *a* and 0, 0.5, 1, 10 and 50 µM for pheophorbide *b*. The Lineweaver–Burk plot was used to determine the inhibition mode. The kinetic parameters were calculated by curve fitting the experimental data to the equation of non-competitive and mixed-type inhibition [11].

### 4.6. Docking Study

PyMOL (Version 2.3.5) (https://pymol.org/2/ accessed on 4 March 2020), Avogadro (Version 1.2.0) (https://avogadro.cc/ accessed on 3 June 2016), AutoDock Tools (Version 1.5.6), and AutoDock vina (Version 1.1.2) (http://vina.scripps.edu/ accessed on 17 December 2020) were downloaded from internet.

The protein structure file was downloaded from Protein Data Bank (Cathepsin K, PDB ID: 6QL8) and the ligand, water molecules, NO_3_ molecules, and amino acids constituting the leader sequence were removed using PyMOL. The compound files ware created using CAS SciFinder^n^ (https://scifinder-n.cas.org/ accessed on 14 January 2021) and the structures were optimized using Gaussian.

Docking study was performed using AutoDock Vina [12,13] without specifying the coupling pocket. The amino acids around the active center (S24, C25, W26, N161, H162) were selected as flexible residues. Finally, the lowest energy pose of the calculated results was selected and visualized with PyMOL.

### 4.7. Inhibition of Cathepsin K by Combination of Allosteric Inhibitors

Inhibition of Cat K activity in the presence of pheophytin *a* or pheophorbide *b* at 5 µM or 0.1 µM, respectively, without or in combination with 10, 20, 50 µM NSC13345 or NSC 94914, was measured in the Cat K inhibition assay as described in Section 4.4.

## Data Availability

The data that support the findings of this study are available from the corresponding author (M.N.) upon reasonable request.

## Supplementary Materials

The following supporting information can be downloaded at: https://www.mdpi.com/article/10.3390/molecules28104197/s1, Section S1: NMR data; Figure S1: Overview of Cat K inhibition assay.

## Sample Availability

The corresponding author (M.N.) may provide samples upon reasonable request.
