# Supplementary material for: Enhancement of Inhibitory Activity by Combining Allosteric Inhibitors Putatively Binding to Different Allosteric Sites on Cathepsin K
Source: Molecules. 2023 May 19;28(10):4197. doi: 10.3390/molecules28104197 (PMC10221639; doi:10.3390/molecules28104197)
Supplement: Supplementary file 1 [file molecules-28-04197-s001.zip › molecules-2340133-supplementary.pdf]

Supplementary Materials

Enhancement of Inhibitory Activity by Combining Allosteric Inhibitors Putatively Binding to Different Allosteric Sites on Cathepsin K

Shun Sato [1], Kana Yamamoto [1], Moeno Ito [1], Katsutoshi Nishino [1], Takanao Otsuka [2], Kazuhiro Irie [3] and Masaya Nagao [1,*]

[1] Graduate School of Biostudies, Kyoto University, Kyoto 606-8502, Japan[2] Department of Applied Chemistry and Biotechnology, Okayama University of Science, Okayama 700-0005, Japan

[3] Graduate School of Agriculture, Kyoto University, Kyoto 606-8502, Japan

* Correspondence: nagao.masaya.7e@kyoto-u.ac.jp; Tel.: +81-75-753-6271

Section S1. NNR data

cassiaoccidentalin B

^{1}H NMR (400 MHz, CD_3OD): δ = 7.42 (1H, d, *J* = 7.2 Hz, H-6′), 7.40 (1H, s, H-2′), 6.92 (1H, d, *J* = 8.5 Hz, H-5′), 6.59 (1H, s, H-3), 6.53 (1H, s, H-6), 5.07 (1H, d, *J* = 8.6 Hz, sugar), 4.04 (1H, d, *J* = 9.7 Hz, sugar), 3.98 (1H, q, *J* = 1.65 Hz, sugar), 3.18 (2H, t, *J* = 9.5 Hz, sugar), 1.45 (3H, d, *J* = 6.0 Hz, sugar).

torachrysone 8-β-gentiobioside

^{1}H NMR (400 MHz, CD_3OD): δ = 7.12 (1H, d, *J* = 2.2 Hz, H-7), 7.07 (1H, s, H-4), 6.87 (1H, d, *J* = 2.2 Hz, H-5), 5.13 (1H, d, *J* = 7.7 Hz, sugar), 4.38 (1H, d, *J* = 7.4 Hz, sugar), 4.21 (1H, d, *J* = 9.4 Hz, sugar), 3.90 (3H, s, H-6) 3.89-3.83 (3H, m, sugar) 3.66-3.61 (1H, m, sugar), 3.57-3.45 (6H, m, sugar), 2.60 (3H, s, H-2), 2.31 (3H, s, H-3).

pheophytin *a*

^{1}H NMR (400 MHz, $CDCl_3$): δ = 9.53 (1H, s, H-10), 9.39 (1H, s, H-5), 8.55 (1H, s, H-20), 8.00 (1H, dd, *J* = 11.5, 17.8 Hz, H-3^1), 6.29 (1H, dd, *J* = 1.4, 17.8 Hz, H-3^2), 6.26 (1H, s, H-13^2), 6.19 (1H, dd, *J* = 1.4, 11.5 Hz, H-3^2), 5.13 (1H, t, *J* = 6.7 Hz, H-p2), 4.52-4.40 (3H, m, H-18, H-p1), 4.21 (1H, d, *J* = 8.8 Hz, H-17), 3.88 (3H, s, H-13^4), 3.69 (3H, s, H-12^1), 3.69 (2H, q, *J* = 7.7 Hz, H-8^1), 3.40 (3H, s, H-2^1), 3.24 (3H, s, H-7^1), 2.65-2.59 (1H, m, H-17^1), 2.52-2.44 (1H, m, H-17^2), 2.39-2.29 (1H, m, H-17^1), 2.23-2.15 (1H, m, H-17^2), 1.80 (3H, d, *J* = 7.3 Hz, H-18^1), 1.69 (3H, t, *J* = 7.7 Hz, H-8^2) 1.87-0.77 (Phytyl group, m), -1.62 (1H, s, H-NH). ^{13}C NMR ($CDCl_3$, 125 MHz) : δ =189.84 (C-13^1), 173.14 (C-17^3), 169.83 (C-13^3), 161.52 (C-16), 156.12 (C-19), 151.28 (C-6), 149.94 (C-14), 149.83 (C-9), 149.00 (C-4), 145.48 (C-8), 143.01 (C-p3), 142.29 (C-1), 138.24 (C-11), 136.79 (C-13), 136.54 (C-3), 136.51 (C-7), 132.08 (C-2), 129.39 (C-3^1), 129.29 (C-12), 122.98 (C-3^2), 118.05 (C-p2), 105.56 (C-15), 104.67 (C-10), 97.80 (C-5), 93.35 (C-20), 64.96 (C-13^2), 61.69 (C-p1), 53.01 (C-13^4), 51.45 (C-17), 50.39 (C-18), 40.02 (C-p4), 39.60 (phytyl group), 37.63 (phytyl group), 37.57 (phytyl group), 37.50 (phytyl group), 36.89 (phytyl group), 33.00 (phytyl group), 32.85 (phytyl group), 31.47 (C-17^2), 30.08 (C-17^1), 29.93 (phytyl group), 28.19 (phytyl group), 25.24 (phytyl group), 24.99 (phytyl group), 24.65 (phytyl group), 23.27 (C-27), 22.91 (phytyl group), 22.83 (phytyl group), 19.94 (phytyl group), 19.87 (phytyl group), 19.71 (C-12^1), 17.60 (C-8^1), 16.50 (C-p3^1), 12.31 (C-2^1), 12.28 (C-8^1), 11.46 (C-7^1).

pheophytin *b*

^{1}H NMR (400 MHz, $CDCl_3$) : δ = 11.09 (1H, s, H-7^1), 10.29(1H, s, H-10), 9.57 (1H, s, H-5), 8.53 (1H, s, H-20), 7.97 (1H, dd, *J* = 11.5, 18.0 Hz, H-3^1), 6.36 (1H, d, *J* = 18.0 Hz, H-3^2), 6.24 (1H, s, H-13^2), 6.22 (1H, d, *J* = 11.5 Hz, H-3^2) 5.15 (1H, t, *J* = 7.2 Hz, H-p2), 4.55-4.40 (3H, m, H-18, H-p1), 4.19 (1H, d, *J* = 8.6 Hz, H-17), 3.97 (1H, q, *J* = 7.7 Hz, H-8^1), 3.91 (3H, s, H-13^4), 3.66 (3H, s, H-12^1), 3.37 (3H, s, H-2^1), 2.67-2.61

(1H, m, H-17^1), 2.55-2.47(1H, m, H-17^2), 2.37-2.30 (1H, m, H-17^1), 2.27-2.20 (1H, m, H-17^2), 1.80 (5H, m, H-18^1, H-8^2), 1.93-0.75 (phytyl group, m), -1.56 (1H, s, H-NH).

pheophorbide *a*

^{1}H NMR (400 MHz, CDCl$_3$): δ = 9.48 (1H, s, H-10), 9.35 (1H, s, H-5), 8.51 (1H, s, H-20), 7.97, (1H, dd, *J* = 18.2, 11.6 Hz, H-3^1), 6.27 (1H, dd, *J* = 18.2, 1.2 Hz, H-3^2), 6.25(1H, s, H-13^2), 6.16 (1H, dd, *J* = 11.8, 1.2 Hz, H-3^2), 4.44 (1H, q, *J* = 7.2 Hz, H-18), 4.19 (1H, d, *J* = 8.6 Hz, H-17), 3.84 (3H, s, H-13^4), 3.66 (2H, q, *J* = 7.6 Hz, H-8^1), 3.65 (3H, s, H-12^1), 3.38 (3H, s, H-2^1), 3.21 (3H, s, H-7^1), 2.65-2.54 (4H, m, H-17^1, H-17^2), 2.34-2.20 (4H, m, H-17^1, H-17^2), 1.80 (3H, d, *J* = 7.2 Hz, H-18^1), 1.67 (3H, t, *J* = 7.6 Hz, H-8^2), -1.63 (1H, s, H-NH).

pheophorbide *b*

^{1}H NMR (500 MHz, CDCl$_3$): δ = 10.90 (1H, s, H-7^1), 10.49 (1H, s, H-10), 9.33 (1H, s, H-5), 8.51 (1H, s, H-20), 7.87, (1H, dd, *J* = 18.3, 11.8 Hz, H-3^1), 6.28 (1H, d, *J* = 18.3 Hz, H-3^2), 6.21(1H, s, H-13^2), 6.16 (1H, d, *J* = 11.8 Hz, H-3^2), 4.44 (1H, q, *J* = 7.4 Hz, H-18), 4.18 (1H, d, *J* = 9.3 Hz, H-17), 3.88 (3H, s, H-13^4), 3.77 (2H, q, *J* = 7.7 Hz, H-8^1), 3.56 (3H, s, H-12^1), 3.32 (3H, s, H-2^1), 2.69-2.60 (2H, m, H-17^1, H-17^2), 2.41-2.35(1H, m, H-17^2), 2.29-2.21 (1H, m, H-17^1), 1.83 (3H, d, *J* = 7.4 Hz, H-18^1), 1.67 (3H, t, *J* = 7.7 Hz, H-8^2), -1.73 (1H, s, H-NH). ^{13}C NMR (CDCl$_3$, 125 MHz) : δ =189.59 (C-13^1), 187.64 (C-7^1), 177.28 (C-17^3), 173.98 (C-19), 169.43 (C-13^3), 159.42 (C-9), 151.08 (C-6), 150.75 (C-14), 147.03 (C-8), 143.64 (C-1), 137.97 (C-11), 137.71 (C-7), 137.14 (C-4), 132.71 (C-3), 132.47 (C-12), 132.30 (C-2), 129.74 (C-13), 128.65 (C-3^1), 123.60 (C-3^2), 106.87 (C-10), 104.96 (C-15), 101.52 (C-5), 93.47 (C-20), 64.68 (C-13^2), 53.16 (C-13^4), 51.37 (C-17), 50.22 (C-18), 31.01 (C-17^1), 29.85 (C-17^2), 23.25 (C-18^1), 19.43 (C-8^2), 18.98 (C-8^1), 12.28 (C-12^1), 12.18 (C-2^1)

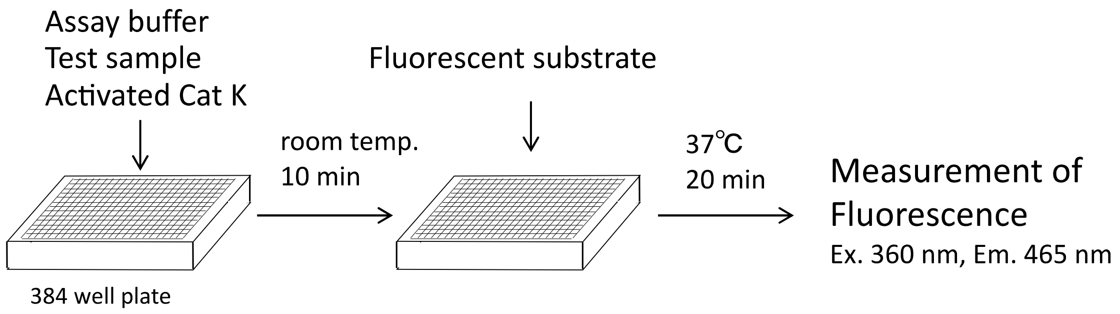

Figure S1. Overview of Cat K inhibition assay.
